# Correlation of Body Mass Index With Severity of Periodontitis: A Cross‐Sectional Study

**DOI:** 10.1002/cre2.70058

**Published:** 2025-03-07

**Authors:** Arvina Rajasekar, Maria Maddalena Marrapodi, Diana Russo, Hande Uzunçıbuk, Vincenzo Ronsivalle, Marco Cicciù, Giuseppe Minervini

**Affiliations:** ^1^ Department of Periodontology, Saveetha Dental College and Hospitals, Saveetha Institute of Medical and Technical Sciences (SIMATS) Saveetha University Chennai Tamil Nadu India; ^2^ Department of Woman, Child, General and Specialist Surgery University of Campania “Luigi Vanvitelli” Naples Italy; ^3^ Multidisciplinary Department of Medical‐Surgical and Odontostomatological Specialties, Oral Surgery Unit University of Campania “Luigi Vanvitelli” Naples Italy; ^4^ Department of Orthodontics, Faculty of Dentistry Trakya University Edirne Turkey; ^5^ Department of Biomedical and Surgical and Dental Sciences University of Catania Catania Italy; ^6^ Saveetha Dental College and Hospitals, SIMATS Saveetha University Chennai Tamil Nadu India; ^7^ Multidisciplinary Department of Medical‐Surgical and Odontostomatological Specialties University of Campania “Luigi Vanvitelli” Naples Italy

**Keywords:** adiposity, body mass index, chronic periodontitis, obesity, risk factors

## Abstract

**Objectives:**

This study aimed to assess the relationship between body mass index (BMI) classification and the severity of periodontitis, recognizing that both obesity and periodontitis involve chronic inflammatory processes, which may exacerbate one another.

**Materials and Methods:**

A cross‐sectional study was conducted on a convenience sample of 162 consecutive outpatients who reported to the Department of Periodontics, Saveetha Dental College and Hospitals in Chennai from March 2023 to September 2023. Age, gender, Russell's periodontal index, and BMI were recorded. The association between age, gender, BMI, and severity of periodontitis was analyzed using linear‐by‐linear *χ* ^2^ association. Ordinal logistic regression analysis was employed to assess the odds ratio (OR) of age, gender, and BMI with the severity of periodontitis.

**Results:**

A statistically significant association was observed between age, gender, BMI, and the severity of periodontitis (*p* < 0.05). Participants aged 35‐60 years had an OR of 1.305 for severe periodontitis (95% CI: 0.754–1.561). Males exhibited a higher risk of severe periodontitis (OR: 1.171; 95% CI: 0.894–2.485). Obese participants showed an OR of 1.417 for severe periodontitis compared to overweight participants (OR: 0.683; 95% CI: 0.817–1.629).

**Conclusions:**

Severe periodontitis was more prevalent among obese individuals, followed by overweight individuals. Obesity may be considered a potential risk indicator for the development and progression of periodontitis.

## 1 Introduction

Obesity is increasing at an alarming rate across the world. It not only makes daily tasks more difficult, but it also doubles the risk of chronic diseases and is also associated with morbidity and mortality. Obesity is a complex condition in which extra body fat has accumulated to the point where it may be harmful to one's health, resulting in increased disease occurrence and shorter life expectancy (Field et al. 2001; Minervini et al. 2023; Di Stasio et al. 2018). Obesity has also been shown to have potentially harmful consequences on the periodontium. Obesity is defined as an excess of body fat in relation to lean body mass (Trayhurn and Wood 2004; Minervini et al. 2018a; d'Apuzzo et al. 2021). Obesity‐related hyperinflammation and cytokine response have been established to be the underlying cause of periodontitis. Obesity increases susceptibility in the host by altering the immunological and inflammatory systems, which predisposes to inflammatory tissue damage and renders the patient at risk for periodontitis. Moreover, adipokines, a class of hormones and cytokines generated by fat tissue, may influence the modulation of periodontitis (Simons et al. 2005; Minervini et al. 2018b; Lo Russo et al. 2023).

Adipokines are polypeptides that are regularly secreted in the adipose tissue. It communicates with other organs, such as the liver, brain, immune system, muscle, and adipose tissue itself in the same way as the traditional circulating hormones. The overexpression of pro‐inflammatory cytokines is caused by changes in the release of these adipokines (Zimmermann et al. 2013; Fukuhara et al. 2005; Marchetti et al. 2012). All of these adipokines play a role in the chronic inflammatory state that is associated with obesity. The development of periodontal disease is accelerated by these pro‐inflammatory mediators.

Periodontitis is a significant dental condition in both developing and developed countries. Periodontitis is defined as a chronic inflammatory disease affecting the supporting tissues of teeth, which is primarily caused by bacterial plaque (Listgarten 1986; Ballini et al. 2020; Inchingolo et al. 2010). Although plaque is the initiating factor, there are a variety of predisposing factors, which include systemic diseases, genetics, hormonal influences, medications, age, gender, smoking, stress, and socioeconomic factors (Rajasekar and Varghese 2022; Rajasekar 2021a, 2021b; Ferguson, Vaid, and Wilcko 2018).

A literature search reveals that the prevalence of periodontitis was high in obese patients and there exists a positive association between obesity and periodontitis (Kongstad et al. 2009; Arboleda et al. 2019; Saito et al. 2001). However, to date, only one study has assessed the relationship between body mass index and the severity of periodontitis (Çetin et al. 2022). Therefore, this study was conducted to assess the relationship of body mass index classification with the severity of periodontitis.

## 2 Materials and Methods

The current cross‐sectional study was carried out at Saveetha Dental College and Hospitals in Chennai. After receiving approval from Saveetha Dental College and Hospitals’ Institutional Ethical Committee (IHEC/SDC/FACULTY/PERIO/351), the study was carried out from March 2023 to September 2023 in compliance with the 2013 revision to the Helsinki Declaration of 1975. Every participant gave their informed consent. G*Power Software, Version 3.0 was used to calculate the sample size based on the mean and standard deviation values from a prior study (Kongstad et al. 2009), and 80% power and a significance level of 0.05 were chosen. A target sample size of 150 was set.

A total of 162 consecutive outpatients who reported to the Department of Periodontics from March 2023 to September 2023 were recruited as study participants. Inclusion criteria were participants aged ≥ 18 years of both genders with a minimum of 20 natural teeth (third molars excluded). Patients with systemic diseases, history of periodontal treatment, tobacco users, pregnant women, and patients on any medications for the past 6 months were all excluded from the study. The participant's age and gender were noted. Assessments of body mass index (BMI) and periodontal health were made. Age, gender, and BMI were independent variables and periodontal status was the dependent variable.

### 2.1 BMI Evaluation

The calculation of BMI involved dividing the weight of each individual in kilograms by the square of their height in meters (kg/m^2^). Based on the BMI, subjects were categorized as follows: normal: BMI of 18.5–24.9, overweight: BMI of 25.0–29.9, and obese: BMI of ≥ 30.0.

### 2.2 Periodontal Examination

Periodontal status was assessed using Russell's Periodontal Index (PI). Based on radiographic evidence of bone loss, pocket formation, tooth mobility, and degree of surrounding tissue inflammation, each tooth was assigned a score (0, 1, 2, 4, 6, 8). Each tooth's score was added up and the total was divided by the total number of teeth that were inspected. The scores were interpreted as follows: Clinically normal supportive tissues were represented by a score of 0–0.2, simple gingivitis by 0.3–0.9, beginning destructive periodontal disease by 1.0–1.9, established destructive periodontal disease by 2.0–4.9, and terminal disease by 5.0–8.0. Based on the interpretation, the participants were categorized as follows: Periodontitis absent: PI score of 0–0.9; Mild periodontitis: PI score of 1.0–1.9; Moderate periodontitis: PI score of 2.0–4.9; and Severe periodontitis: PI score of 5.0–8.0.

### 2.3 Statistical Analysis

The Statistical Package for Social Sciences (SPSS Software, Version 23.0; IBM Corp., Armonk, NY, USA) was used to analyze the data. When the *p*‐value was less than 0.05, the results were deemed statistically significant. The characteristics of participants were assessed using frequency distribution. Periodontitis prevalence was estimated using the frequency distribution in relation to age, gender, and BMI. The association of age, gender, and BMI with the severity of periodontitis was analyzed by a linear‐by‐linear *χ* ^2^ association. Ordinal logistic regression was utilized to find the risk of age, gender, and BMI with periodontitis at a 95% confidence interval.

## 3 Results

A total of 162 participants with ages ranging from 18 to 60 years were enrolled in the present study. Table 1 summarizes the sociodemographic characteristics, BMI of the participants, and prevalence of periodontitis. Based on the characteristics of the participants, 53.1% of the study subjects belonged to the age group of 35‐60 and 54.9% were females. Based on BMI, 66 participants (40.7%) belonged to the obese category followed by 59 participants (36.4%) in the overweight category, and 37 (22.8%) of them presented with normal weight. Also, according to the severity of periodontitis, participants with mild, moderate, and severe periodontitis were 42 (25.9%), 26 (16%), and 43 (26.5%), respectively.

**Table 1:** Frequency distribution of age, gender, BMI, and periodontitis among the study participants.

Variable	Sub‐variable	Frequency	Percentage
Age	18–34 years	76	46.9
	35–60 years	86	53.1
Gender	Male	73	45.1
	Female	89	54.9
BMI	Normal	37	22.8
	Overweight	59	36.4
	Obese	66	40.7
Periodontitis	Absent	51	31.5
	Mild	42	25.9
	Moderate	26	16.0
	Severe	43	26.5

Table 2 shows the frequency distribution of periodontitis. On comparing different age groups, participants of 35–60 years showed a higher prevalence of severe periodontitis (33.7%) as compared to 18–34 years of age (18.4%). On gender‐wise comparison, a higher proportion of males presented with severe periodontitis (41.1%) than females (14.6%). Based on BMI, severe periodontitis was observed predominantly among obese patients (43.9%) followed by overweight individuals (23.7%).

**Table 2:** Frequency distribution of periodontitis among different age groups, gender, and BMI.

Variables	Periodontitis, *n* (%)			
	Absent	Mild	Moderate	Severe
Age group				
18–34 years	32 (42.1)	21 (27.6)	9 (11.8)	14 (18.4)
35–60 years	19 (22.1)	21 (24.4)	17 (19.8)	29 (33.7)
Gender				
Male	13 (17.8)	14 (19.2)	16 (21.9)	30 (41.1)
Female	38 (42.7)	28 (31.5)	10 (11.2)	13 (14.6)
BMI				
Normal	26 (70.3)	11 (29.7)	—	—
Overweight	13 (22)	17 (28.8)	15 (25.4)	14 (23.7)
Obese	12 (18.2)	14 (21.2)	11 (16.7)	29 (43.9)

Table 3 depicts the association of independent variables with the severity of periodontitis. A linear‐by‐linear *χ* ^2^ association was considered since the table is not a contingency table. The linear‐by‐linear *χ* ^2^ association showed a significant association of age, gender, and BMI with the severity of periodontitis (*p* < 0.05). On the significant association of independent variables with the dependent variable, ordinal logistic regression was performed to find the risk of independent variables with periodontitis.

**Table 3:** Cross‐tabulation for association of age, gender, and BMI to periodontitis.

Independent variable	Count	Dependent variable				Linear‐by‐linear *χ* ^2^ association	*p* value
		Absent	Mild	Moderate	Severe		
Age							
18–4 years	Actual	32	21	9	14	9.847	0.002
	Expected	23.9	19.7	12.2	20.2		
35–60 years	Actual	19	21	17	29		
	Expected	27.1	22.3	13.8	22.8		
Gender							
Male	Actual	13	14	16	30	22.399	0.000
	Expected	23	18.9	11.7	19.4		
Female	Actual	38	28	10	13		
	Expected	28	23.1	14.3	23.6		
BMI							
Normal	Actual	26	11	0	0	37.837	0.000
	Expected	11.6	9.6	5.9	9.8		
Overweight	Actual	13	17	15	14		
	Expected	18.6	15.3	9.5	15.7		
Obese	Actual	12	14	11	29		
	Expected	20.8	17.1	10.6	17.5		

Ordinal logistic regression was considered as the dependent variable was an ordinal type of variable (mild, moderate, and severe). To proceed with ordinal logistic regression, an important assumption of non‐multicollinearity (association between independent variables) should be obtained. Testing for multicollinearity showed a VIF of 1.007, 1.007, and 1.000, which indicates no association between independent variables (age, gender, and BMI). Thus, ordinal logistic regression was done on writing syntax in the software with a high model fit using Negelkerke *R* ^2^. The model fit was found to be good for all independent variables. Considering the age group, participants who belonged to 35‐60 years had 1.305 odds for severe periodontitis with 95% CI (0.754–1.561). Similarly, compared to females, males had higher odds of risk for severe periodontitis (1.171) with 95% CI (0.894–2.485). Obese participants had 1.417 odds of risk for severe periodontitis compared to overweight participants (0.683) with 95% CI (0.817–1.629) (Table 4).

**Table 4:** Ordinal logistic regression showing the magnitude of risk for severe periodontitis.

Characteristics	Model fit (Negelkerke *R* ^2^)	Exp *β* (odd's ratio)	*p* value	95% confidence interval
BMI				
Normal	47.4%	1	—	—
Overweight		0.683	0.000	0.011–0.080
Obese		1.417	0.011	0.817–1.629
Age				
18–34 years	43.3%	1	—	—
35–60 years		1.305	0.000	0.754–1.561
Gender				
Female	21.5%	1	—	—
Male		1.171	0.000	0.894–2.485

## 4 Discussion

The current research aimed to assess the relationship of body mass index classification with the severity of periodontitis. In the present study, the prevalence of severe degree of periodontitis was high and statistically significant among obese individuals as compared to overweight and normal weight individuals. Periodontal disease was shown to be prevalent among obese populations, affecting 74% of Malaysians (Khan et al. 2015), 51.9% of Jordanians (Al‐Zahrani, Bissada, and Borawski 2003), and 35% of Americans (Khader et al. 2009). Similarly, Alsalihi et al. found that obese patients in Bahrain had a significant prevalence of periodontitis (Alsalihi et al. 2021). These investigations revealed that the prevalence of periodontal disease among obese people has been rising in comparison to normal weight people. Furthermore, the present study highlighted that the obese participants had an increased risk for periodontitis as compared to overweight and normal weight participants. Bhardwaj et al. suggested that each 1 kg/m^2^ increase in BMI increased the risk of periodontitis by 56% (Bhardwaj et al. 2013). Another study reported that obese patients had 1.57 odds of risk for acquiring periodontitis (Gul et al. 2021). Our results are in agreement with the prior findings. This could be due to the upregulation of inflammatory response and downregulation of immune response in obesity, which in turn leads to the progression of periodontal disease.

Furthermore, the present study documented that the older the subjects, the higher the prevalence of periodontitis, and its influence was statistically significant as the prevalence of severe periodontitis was high among 35–60‐year‐old patients. Clinical attachment loss increases after 40 years, according to the observations of the National Health and Nutrition Examination Survey (Jiao et al. 2020). A study by Wulandari P et al. reported that the periodontitis prevalence was higher among the 40–60 age group as compared to other age groups and the severity of periodontitis showed a statistically significant correlation with age (Wulandari et al. 2022; Dib Zakkour et al. 2024). A similar association was observed in various other studies (Machado et al. 2018; Huttner et al. 2009). It could be speculated by the fact that age is an established risk determinant for periodontitis. As age advances, the exposure to bacterial biofilm increases, and also certain physiological changes that occur in the connective tissues predispose the periodontium to break down over time. Also, a decrease in bone quality is a part of aging. Bone constantly renews itself through a process known as remodeling, where older bone tissue is broken down and replaced with new bone. This activity takes place in specific regions called bone metabolic units. In these units, the actions of osteoblasts, which form bone, and osteoclasts, which resorb bone, are closely coordinated to ensure a balance that maintains bone density and structural integrity. However, as individuals age, this equilibrium shifts unfavorably, leading to increased bone resorption and decreased bone formation, ultimately compromising bone quality (Rutger Persson 2006; Naidu et al. 2024). This impacts the periodontal health negatively.

When the association between gender and severity of periodontitis was assessed, this study found that males presented with an increased risk for periodontitis. According to Al‐Alimi et al. the male gender was linked to the highest risk of periodontitis (Al‐Alimi et al. 2018). Similarly, when comparing men and women, more men had moderate to severe periodontal destruction than women, and the difference was statistically significant (Mamai‐Homata, Koletsi‐Kounari, and Margaritis 2016). The same findings were observed in previous studies (Stănescu et al. 2020; Sanadi et al. 2017). This gender disparity could be explained by the negligence of oral health by males and men are less likely than women to seek preventive dental care. According to the 2007 National Ambulatory Medical Care Survey, approximately 60% of men postpone dental care services even during serious illness (National Ambulatory Medical Care Survey, 1981; [Internet] 1985; Alshahrani et al. 2024; Scribante, Gallo, and Pascadopoli 2024). In addition, men are more likely than women to use tobacco‐related products, which has negative consequences for periodontal health (Genco and Borgnakke 2013; Fiorillo et al. 2024; Nery et al. 2024).

A few limitations present in this study, which include the cross‐sectional study design and the samples, do not represent a diverse population. Furthermore, variations in oral hygiene practices among the study participants present an additional limitation. Also, the present study considered only BMI to define obesity. Further studies are needed considering other indicators of obesity, such as waist‐hip ratio, waist circumference, and body fat percentage to correlate with the periodontal parameters. However, the present study is unique in its way as the majority of the previous studies looked into either the prevalence of periodontitis among obese and normal weight subjects or the severity of periodontitis among the obese population. In contrast with a broad idea, the present study investigated the correlation between the body mass index classification with the severity of periodontitis. Furthermore, this study assessed the correlation of other risk determinants including age and gender with the severity of periodontitis. Also, another strength of this study is that the periodontal status was assessed using Russell's Periodontal Index scores, which is based on full mouth periodontal examination rather than assessing the selected teeth, thereby providing the definitive grading of periodontitis.

## 5 Conclusion

Within the limitations, the present study suggests that severe periodontitis was more prevalent among obese followed by overweight individuals. Therefore, obesity could be a potential risk indicator for periodontitis. In addition, the study showed that periodontitis severity was influenced by age and gender. However, age, gender, and BMI‐matched case‐control studies are warranted to confirm the association of these factors with periodontitis. Therefore, this research highlights the importance of educating obese people about the potential oral implications of obesity to avoid chronic illness. Also, the clinical landscape must evolve to address the burgeoning epidemic of periodontal disease among obese individuals. Routine periodontal health screening can serve as an invaluable tool to detect early signs of gingivitis or periodontitis, allowing for timely intervention. By incorporating such screenings into standard practice, healthcare professionals can foster a multidisciplinary approach by referring patients to periodontists, as needed.

## Author Contributions

All authors have contributed significantly to this work. Arvina Rajasekar and Giuseppe Minervini conceptualized and designed the study. Arvina Rajasekar was responsible for data collection and statistical analysis. Maria Maddalena Marrapodi, Diana Russo, and Hande Uzunçıbuk contributed to data interpretation and manuscript drafting. Vincenzo Ronsivalle, Marco Cicciù, and Giuseppe Minervini critically revised the manuscript for important intellectual content. All authors read and approved the final manuscript.

## Ethics Statement

This study was approved by the Saveetha Dental College and Hospitals' Institutional Ethical Committee (IHEC/SDC/FACULTY/PERIO/351).

## Consent

The authors have nothing to report.

## Conflicts of Interest

The authors declare no conflicts of interest.

## Data Availability

The data that support the findings of this study are available from the corresponding author upon reasonable request.
